# Association of CYP2J2 polymorphism with susceptibility to psoriasis in Turkish population: a case–control study^☆^^☆☆^

**DOI:** 10.1016/j.abd.2019.04.006

**Published:** 2019-12-10

**Authors:** Yıldız Hayran, Nuran Allı, Pınar İncel Uysal, Tuba Çandar

**Affiliations:** a Department of Dermatology, Ankara Numune Training and Research Hospital, Ankara, Turkey; b Department of Medical Biochemistry, Ufuk University Faculty of Medicine, Ankara, Turkey

**Keywords:** Disease susceptibility, Psoriasis, Polymorphism, Single nucleotide

## Abstract

**Background:**

Cytochrome P450 2J2 is mostly expressed in extrahepatic tissues; it metabolizes arachidonic acid to epoxyeicosatrienoic acids, with various cardio protective and anti-inflammatory effects. CYP2J2 polymorphism has been identified as a risk factor for cardiovascular diseases, but its association with psoriasis remains unknown.

**Objective:**

To evaluate CYP2J2 polymorphism as a risk factor for psoriasis in the Turkish population.

**Methods:**

There were 94 patients with psoriasis and 100 age- and sex-matched healthy controls included in the study. Detailed demographic and clinical characteristics were recorded, and Psoriasis Area and Severity Index (PASI) scores were calculated for psoriasis patients. Venous blood samples were collected from all the participants and CYP2J2 50G>T (rs890293) polymorphism was analyzed using polymerase chain reaction (PCR).

**Results:**

Both T allele and TT + GT genotype frequencies were increased in psoriasis vulgaris patients compared to the control group (*p* = 0.024 and *p* = 0.029 respectively, OR = 2.82, 95% CI: 1.11–7.15) No association between CYP2J2 polymorphism and clinical features of psoriasis was identified.

**Study limitations:**

A limited number of patients were included in the study.

**Conclusion:**

CYP2J2 50G>T (rs890293) polymorphism was associated with an increased risk for PsV in the Turkish population.

## Introduction

Psoriasis is a chronic inflammatory skin disease characterized by sharply demarcated erythematous papules and plaques with silvery scales. However, the prevalence varies widely between studies (0–11.8%) according to age, gender, and race; psoriasis is thought to affect about 2% of the general population.^1^ Psoriasis affects men and women equally. Although it can start at any age, two age peaks in the incidence have been observed: the first in second and third decade, and the second in fifth and sixth decade.^2^ Skin, nail, or joint involvement can be seen in up to 50% of the patients, and the association of psoriasis with many systemic disorders such as cardiovascular diseases, infections, metabolic disorders, kidney diseases, gastrointestinal diseases, and malignancies is well documented.^3^, ^4^, ^5^ The etiopathogenesis of the disease is not well understood. Psoriasis is categorized as a multifactorial disease with genetic, environmental, immunological, and inflammatory factors that contribute to pathogenesis of the disease.^6^ Inflammation, both lesional and systemic, is an important feature of psoriasis. Many studies investigating systemic inflammation in psoriasis revealed that markers of inflammation (especially C-reactive protein, tumor necrosis factor [TNF]-α, intracellular adhesion molecule [ICAM]-1, E-selectin, and interleukin [IL]-1β, IL-6, IL-10) are increased in psoriatic patients, creating a systemic inflammatory state that could lead to comorbidities like cardiovascular diseases.^7^

Cytochrome P450 (CYP) enzymes are hemoproteins mostly located in the endoplasmic reticulum (ER) or mitochondrial membrane in humans. The main function of CYPs is to metabolize drugs, non-drug xenobiotics, and endobiotics such as fatty acids, steroids and steroid hormones, bile acids, Vitamin D, prostaglandins, and arachidonic acid (AA).^8^, ^9^, ^10^ AA is metabolized to four epoxyeicosatrienoic acids (EETs) 5,6-EET, 8,9-EET, 11,12-EET, and 14,15-EET. EETs promote vascular relaxation, have an anti-inflammatory effect in endothelium, and a cardioprotective effect during ischemic events.^11^, ^12^ Besides their favorable effect on cardiovascular and inflammatory diseases, EETs may also promote proliferation, migration, and survival in tumor cells.^13^ CYP2J2 – CYP isoform mostly expressed in the liver, heart, small intestine, kidney, and brain – is one of the main CYPs converting AA to EETs.^14^ CYP2J2 polymorphism has been investigated in cardiovascular diseases, ischemic stroke, and Alzheimer's disease, and it has been shown to increase susceptibility to these diseases.^15^, ^16^, ^17^, ^18^

The aim of this study was to investigate CYP2J2 polymorphism in psoriasis, a chronic inflammatory skin disease that can be accompanied by cardiovascular diseases.

## Materials and Methods

### Study population

In the present study, 94 clinically or histopathologically diagnosed psoriasis patient admitted to the Ankara Numune Training and Research Hospital between January 2015 and July 2016. As a control group, 100 age and sex-matched patients admitted to the dermatology outpatient clinic with a dermatological disease other than psoriasis – with no history of any chronic inflammatory disease – were recruited. Demographic characteristics of both psoriasis patients and the control group, and clinical features (disease subtype, duration, severity, family history, treatments, history of psoriatic arthritis, and involvement of special localization such as the scalp, flexural areas, and nails) of the disease were recorded. Psoriatic arthritis was investigated in all patients. History of psoriatic arthritis was noted and patients with active arthralgia or arthritis were referred to the rheumatology department for a definitive diagnosis of psoriatic arthritis. Children (age < 18), pregnant and breastfeeding participants, and participants with history of any immunological or inflammatory diseases were excluded from the study.

The study was approved by local ethics committee (E-15-612) and all participants gave written informed consent prior to the study.

### Polymorphism analysis

Primer design was carried out for the polymerase chain reaction (PCR) amplification of the CYP2J2-50G>T polymorphism region, so that the locus of the polymorphism was at a proximity of 150 bp from both ends of the PCR amplicon. This is because a sequencing kit was used, which reads 150 bp from both ends. Before the primers were ordered, the authors added specific sequences of about 30 nucleotides to 5′ ends of the primers, to make the amplicons suitable for indexed sequencing on Illumina platforms. Table 1 summarizes PCR primer pairs, locus specific sequences, and sequences added for index PCR. DNA samples were obtained with the isolation from 200 μL blood samples from each individual, by using a QIAamp DNA Blood Mini Kit (Qiagen Inc.). PCRs were carried out on isolated DNA samples by using the designed primers and the reactions were checked by using 2% agarose gel electrophoresis. A second PCR for indexing purposes was conducted to add index sequences to the amplicons, in order to achieve multiplexing in next-gene sequencing; for each of the samples, a different combination of index primers was used. Indexing PCR reactions were also checked by using 2% agarose gel electrophoresis. Equal volumes of indexed PCR reactions were mixed to obtain a PCR pool, which had all the amplicons of all samples. The PCR pool was purified by using a NucleoFast® 96 PCR kit (Macherey-Nagel GmbH). The purified pool was quantified by using a micro volume spectrophotometer and diluted according to the recommendations of Illumina Inc. Next-gene sequencing of the samples was carried out by using the Miseq system (Illumina Inc.). Genotyping of the samples was achieved as the data was analyzed on IGV v. 2.3 software (Broad Institute).

**Table 1:** PCR primer pairs, locus-specific sequences, and sequences added for PCR index.

*Primers*	
CYP2J2_-50_F	TCGTCGGCAGCGTCAGATGTGTATAAGAGACAGCTCCTAGCCTGGCCTTTTCTGAGAC
CYP2J2_-50_R	GTCTCGTGGGCTCGGAGATGTGTATAAGAGACAGCATGGCTCAGACGTCCTCCTGCTC

*Locus-specific sequences*	
CYP2J2_-50_F	CTCCTAGCCTGGCCTTTTCTGAGAC
CYP2J2_-50_R	CATGGCTCAGACGTCCTCCTGCTC

*Sequences added for index PCR*	
seq (i5) + adapter In constructed primer	TCGTCGGCAGCGTCAGATGTGTATAAGAGACAG
seq (i7) + adapter in constructed primer	GTCTCGTGGGCTCGGAGATGTGTATAAGAGACAG

### Statistical analysis

SPSS Statistics for Windowsv. 21.0 (IBM Corp. – Armonk, NY, United States) was used for statistical analysis. Quantitative variables were analyzed using the chi-Squared test or Fisher's exact test where needed, and qualitative variables were analyzed using the Mann–Whitney *U* test. Correlation between qualitative variables was assessed using Spearman's correlation test. The statistically significant difference was predetermined as *p* < 0.05 for all analyses.

## Results

### Patient characteristics and clinical features of psoriasis

Patient characteristics and clinical features of psoriasis are summarized in table 2. Forty-seven percent of the patients were male and 53% were female. The mean age of patients was 44.66 (SD = 12.6) and mean age of psoriasis diagnosis was 33.21 (SD = 14.9). The age of patients and age of diagnosis were similar in both male and female patients (*p* = 0.39 and 0.53, respectively).

The mean PASI of psoriasis patients was calculated as 4.51 (SD = 3.86). Psoriasis vulgaris was the most common type of psoriasis and was identified in 76% of all psoriasis patients. Palmoplantar psoriasis was the second most common type of psoriasis (11%), followed by guttate psoriasis (6%), erythrodermic psoriasis (4%), and pustular psoriasis (3%). Involvement of at least one of the special locations such as the scalp, flexural region, or genital region was identified in 74.5% of psoriasis patients. Scalp involvement was most frequently affected special location. Arthralgia was identified in 47% of psoriasis patients and 23% of patients were diagnosed with psoriatic arthritis.

Previous and treatment treatments are summarized in table 2. Topical steroids were the most common previous and current treatment of psoriasis patients (99% and 90%, respectively). Among the patients, 23.1% had at least one associated systemic disease or comorbidity. Cardiovascular diseases were present in 11.7% of the patients and other systemic diseases were present in 22.2% (Table 2).

**Table 2:** Patient characteristics and clinical features of psoriasis.

Characteristic	*n* (%)
*Age (years)*	45 (36–54)^a^
*Sex men,*	45 (47.9)

*Clinical type*	
Psoriasis vulgaris	71 (75.5)
Non-vulgar psoriasis	23 (24.5)
Age of psoriasis diagnosis	31 (21–46)^a^
PASI	4.2 (1.85–6.9)^a^

*Presence of involvement of special locations*	
Scalp	57 (60.6)
Nail	31 (33)
Flexural region	24 (25.5)
Genital region	21 (22.3)
Presence of arthralgia	46 (48.9)
Presence of arthritis	23 (24.5)
Positive family history for psoriasis	26 (7.7)

*Previous treatments*	
Topical steroids	93 (98.9)
Systemic treatments	52 (55.3)
Phototherapy	3 (3.2)
Biologics	2 (2.1)

*Current treatments*	
Topical steroids	85 (90.4)
Systemic treatments	39 (41.5)
Phototherapy	12 (12.8)
Biologics	21 (22.3)

*Associated internal diseases and comorbidities*	
Cardiovascular diseases	11 (11.7)
Other systemic diseases	22 (22.2)

a Data presented as median (IQR). PASI, Psoriasis Area and Severity Index.

### CYP2J2 gene polymorphism analysis

Allele frequency and genotype distribution of CYP2J2 gene in psoriasis patients and controls are summarized in table 3. T allele and TT + GT genotype in the dominant model were more frequent in psoriasis patients compared to controls (T allele frequency 9.04% *vs.* 4.5% and TT + GT genotype frequency 17.02% *vs.* 8%) but this clinical difference did not reach a statistically significant level (*p* = 0.063 and *p* = 0.062, respectively). Psoriasis patients were divided into two subgroups as psoriasis vulgaris (PsV) and non-vulgar psoriasis (nVP). Subgroup analysis showed that both T allele and TT + GT genotype frequencies were increased in PsV patients compared to control group (*p* = 0.024 and *p* = 0.029, respectively), indicating that CYP2J2 polymorphism was associated with increased risk of developing PsV (OR = 2.82, 95% CI: 1.11–7.15, *p* = 0.029).

**Table 3:** Allele frequency and genotype distribution of CYP2J2 gene in psoriasis patients and controls.

		Psoriasis (all forms) (n = 94) n (%)	PsV (n = 71) n (%)	Control (n = 100) n (%)	p-value (psoriasis [all forms] *vs.* control)	p-value (PsV *vs.* control)	OR (95% CI) (psoriasis [all forms] *vs.* control)	OR (95% CI) (PsV *vs.* control)
*Genotype*								
Dominant model	TT + GT	16 (17.02)	14 (19.8)	8 (8)	0.062	0.029	2.35 (0.96–5.8)	2.82 (1.11–7.15)
	GG	78 (82.97)	57 (80.2)	92 (92)				
Recessive model	TT	1 (1.06)	1 (1.4)	1 (1)	0.96	0.807	1.07 (0.07–17.3)	1.41 (0.09–22.9)
	GT + GG	93 (98.94)	70 (98.6)	99 (99)				
*Allele*								
	G	171 (90.96)	127 (89.5)	191 (91.2)	0.063	0.024		
	T	17 (9.04)	15 (10.5)	9 (4.5)				

PsV, psoriasis vulgaris.

Demographic features and disease characteristics of psoriasis patients with GG genotype (wild type) and GT + TT genotype (homozygous or heterozygous polymorphism) were compared and summarized in table 4. Although patients with PsV are more frequent than patients with nPV in polymorphic group (>2-fold), the result wasn’t statistically significant (*p* = 0.22). Age and sex of the patients, age of onset, PASI, presence of involvement of special locations, presence of arthralgia and arthritis, positive family history for psoriasis, presence associated internal diseases, and treatment modalities were similar between psoriasis patients with and without polymorphism.

**Table 4:** Demographic features and disease characteristics of psoriasis patients with GG genotype and GT + TT genotype.

	CYP2J2 GG	CYP2J2 GT + TT	*p*-Value
*Psoriasis type (PsV/nVP)*	57/21	14/2	0.22
*Age*	44.6 (10.9)	46 (15.9)	0.90
*Age ofonset*	30 (13.7)	31 (16.1)	0.41
*PASI*	4.5 (4)	5.2 (2.8)	0.81
*Sex, male/female*	27/29	7/8	0.92

*Presence of involvement of special locations*			
Scalp	66.1	73.3	0.59
Nail	37.5	26.7	0.44
Flexural region	26.8	26.7	0.99
Genital region	25	13.3	0.34

*Presence of arthralgia*	50	53.3	0.82
*Presence of arthritis*	21.4	26.7	0.67
*Positive family history for psoriasis*	35.7	20	0.25
*Presence associated internal diseases and comorbidities*	26.8	20	0.59

## Discussion

This study investigated the possible role of CYP2J2 polymorphism as a risk factor in psoriasis patients. The results demonstrate a significantly higher frequency of T allele in psoriasis vulgaris patients and an increased risk of psoriasis in patients with CYP2J2 polymorphism in the dominant model. To the best of the authors knowledge, this is the first study investigating the role of CYP2J2 polymorphism in psoriasis.

CYP2J2 50G>T is one of the most studied functional CYP2J2 polymorphisms. Spiecker et al. studied the effect of CYP2J2 50G>T polymorphism and observed a decrease in binding of transcription factor Sp1 to DNA, a reduction in promoter activity, and lower EET plasma concentrations in patients with G-50T polymorphism compared with wild type participants.^19^ CYP2J2-derived EETs induce vasodilatation; inhibit inflammation, apoptosis, and thrombosis; and show cardio-protective effects.^20^ By altering their plasma concentrations, CYP polymorphism may affect the cardio-protective role of EETs and play a role in the pathogenesis of cardiovascular diseases.

CYP2J2 polymorphism has been studied mainly in cardiovascular diseases, with contradictory results among different ethnic groups. Studies investigating CYP2J2 polymorphism in coronary artery diseases revealed an increase risk in the German population, decreased risk in African Americans, and no association in Caucasians and the Swedish population.^18^, ^19^, ^21^, ^22^ Polonikov et al. showed an increased risk of hypertension in Russian patients; however, this association couldn’t be confirmed in African Americans or Caucasians.^23^, ^24^, ^25^ Polonikov et al. also described a sex-specific relationship between CYP2J2 polymorphism and hypertension in female Russian patients.^26^ No significant association between CYP2J2 polymorphism and stroke was identified in Swedish and Chinese populations.^18^, ^27^ Recent studies also demonstrated that CYP2J2 increased circulating EET levels, induced angiogenesis, and improved cardiac function in rats after myocardial infarction.^28^ Cardiovascular diseases are frequent and important comorbidities in psoriasis. The present study evaluated the association between CYP2J2 polymorphism and cardiovascular comorbidities. No significant association was observed between CYP2J2 polymorphism and comorbidity frequency, suggesting that CYP2J2 may increase the risk of developing psoriasis vulgaris, but that it is not associated with an increased risk of cardiovascular comorbidities in psoriasis patients.

Besides its cardiovascular effects, CYP2J2-derived EETs also modulate inflammation.^29^ Node et al. studied the effect of CYP2J2-derived EETs on vascular inflammation. Their study revealed that EETs may modulate inflammatory response by inhibition of pro-inflammatory cytokine-induced (TNF-α, IL-1α, and bacterial lipopolysaccharide) expression of vascular endothelial adhesion molecules (VCAM-1, ICAM-1, and E-selectin) and decrease the number of rolling/adherent mononuclear leucocytes.^29^ EETs also show their anti-inflammatory effects through inhibition of nuclear factor-kappaB (NF-κB) and IκB kinase, activation of peroxisome proliferator-activated receptors, increase of eNOS expression, and regulation of endoplasmic reticulum homeostasis.^30^

Although the anti-inflammatory effect of EETs is well documented, there are only a few studies investigating CYP2J2 polymorphism in inflammatory diseases. Wang et al. studied CYP2J2 polymorphism in diabetes mellitus patients. Although no significant difference was observed between diabetic patients and a control group; among diabetic patients, CYP2J2 polymorphism was shown to be associated with younger age of onset.^31^ The present authors studied CYP2J2 polymorphism in psoriasis, a chronic inflammatory skin disease, and revealed an increased risk of psoriasis in patients with CYP2J2 polymorphism. In the pooled analysis where all psoriasis patients were included, although CYP polymorphism was higher in psoriasis patients, the results did not reach statistical significance. In the subgroup analysis of psoriasis subtypes, a statistically significant difference between CYP polymorphism of psoriasis vulgaris patients and the control group was observed. CYP polymorphism was identified as a risk factor for PV but not for nVP. Psoriasis is an inflammatory skin disorder in which genetic susceptibility has a significant effect on pathogenesis of the disease. Family history of psoriasis differs among ethnic groups and the prevalence of a positive family history is estimated at approximately 30%.^32^, ^33^ PSORS1 and PSORS2 are two of the best known susceptibility loci for psoriasis. HLA-C*06:02 is located at the PSORS1 locus and the role of HLA-C*06:02 in psoriasis is well known. Recent studies confirmed the importance of HLA-C*06:02 in genetic susceptibility of psoriasis along with its effect on treatment responses.^34^ Together with HLA-C*06:02, a large genetic study conducted by Zhou et al. revealed several new susceptibility loci, such as HLA-C*07:04, rs118179173, HLA-B amino acid 67, HLA-DPB1*05:01, and BTNL2 amino acid 281.^35^ Caspase recruitment family member 14 (CARD14) is located at PSORS2 locus and activates the NF-κB pathway. CARD14 has been classified as a psoriasis susceptibility gene and many recent studies have focused on demonstrating the role of CARD14 in the pathogenesis of psoriasis. Gain-of-function mutations of CARD14 activate the NF-κB signal transduction pathway, and increased NF-κB activity due to CARD14 mutations is shown to be linked to pustular psoriasis.^36^ Zhu et al. also investigated CARD14 variants in patients with psoriasis vulgaris. The study revealed five mutations, but the mutations were also seen in the control group and a clear mutation-disease relationship could not be defined.^37^ Tanaka et al. investigated the immunological role of CARD14 in psoriasis murine model and showed that IL23 mRNA expression and IL-17 producing T cell infiltration was decreased in CARD14-deficient mice.^38^ The study also showed that psoriasiform inflammatory skin changes were decreased in CARD14-deficient mice, suggesting the possible immune inflammatory role of CARD14 in psoriasis pathogenesis.^38^ Besides PSORS1 and PSORS2, many psoriasis susceptibility loci were identified with genome-wide association studies (GWAS). Tsoi et al. performed a GWAS meta-analysis and identified 16 new susceptibility loci that regulate the I-κB kinase/NF-κB pathway and cytotoxicity, and that respond to external stimuli and leukocyte differentiation.^39^ In addition to these genetic variations some subtype specific mutations and polymorphisms were also identified. Li et al. showed that polymorphism in the IL-36 receptor antagonist gene (IL36RN) was associated with increased risk of pustular psoriasis.^40^ Twelves et al. investigated the IL36RN mutation frequencies between different pustular psoriasis subtypes and revealed that IL36RN mutations were more frequent among patients with generalized pustular psoriasis.^41^ Studies showing different genetic background in different psoriasis subtypes suggest that non-vulgar psoriasis may be a different disease rather than a subtype of psoriasis, which is consistent with the present results.

The results showed no association between CYP2J2 polymorphism and clinical characteristics of psoriasis. The psoriasis patients included in the study were mostly under treatment; with treatment, major clinical characteristics such as PASI, nail involvement, arthralgia, and arthritis could be modified.

One of the limitations of this study is that it did not study the EET concentrations and their association with CYP2J2 polymorphism. CYP2J2 polymorphism may contribute to inflammatory process and pathogenesis of psoriasis through altering ETT concentrations, but to understand the psoriasis-CYP polymorphism-ETT relationship better, further studies are needed. Another limitation is the limited number of psoriasis patients with cardiovascular comorbidities. We did not show any relationship between CYP polymorphism and comorbidities, but this could be due to type II (beta) error.

The genetic basis of psoriasis is complex and differs among ethnic groups as well as psoriasis subtypes. CPY polymorphism – like other polymorphisms – may vary among ethnic groups. In order to generalize these results further studies of different ethnic groups and different psoriasis subtypes are needed.

## Financial support

This study was supported by a research grant from the Turkish Society of Dermatology (2017/53).

## Authors’ contribution

Yıldız Hayran: Statistical analysis; approval of the final version of the manuscript; conception and planning of the study; composition of the manuscript; collection, analysis, and interpretation of data; participation in the design of the study; intellectual participation in the propaedeutic and/for therapeutic conduct of the studied cases; critical review of the literature; critical review of the manuscript.

Nuran Allı: Approval of the final version of the manuscript; critical review of the manuscript.

Pınar İncel Uysal: Composition of the manuscript; collection, analysis, and interpretation of data.

Tuba Çandar: Participation in the design of the study; critical review of the manuscript.

## Conflicts of interest

None declared.
